# Repeatability and reproducibility of retinal and choroidal thickness measurements in Diabetic Macular Edema using Swept-source Optical Coherence Tomography

**DOI:** 10.1371/journal.pone.0200819

**Published:** 2018-07-26

**Authors:** Anna Sala-Puigdollers, Marc Figueras-Roca, Mireia Hereu, Teresa Hernández, Montse Morató, Alfredo Adán, Javier Zarranz-Ventura

**Affiliations:** 1 Institut Clínic d'Oftalmologia (ICOF), Hospital Clínic, Barcelona, Spain; 2 August Pi i Sunyer Biomedical Research Institute (IDIBAPS), Barcelona, Spain; 3 Institut de la Màcula, Centre Mèdic Teknon, Barcelona, Spain; LV Prasad Eye Institute, INDIA

## Abstract

**Purpose:**

To evaluate the repeatability and reproducibility of retinal and choroidal thickness measured with Swept source Optical Coherence Tomography (SS-OCT) in eyes with Diabetic Macular Edema (DME).

**Methods:**

42 DME eyes were imaged using SS-OCT standard Macular scanning protocols. Retinal and choroidal thickness were measured in the Total macular circle (TMC) and foveal central subfield (FCS) using device-integrated specific software. The coefficient of repeatability (CR) and intraclass correlation coefficient (ICC) were determined as a measure of repeatability and relative reliability within graders. Reproducibility was assessed using Bland-Altman plots and 95% limits of agreement (LoA) were determined as a measure of interobserver variability.

**Results:**

Intragrader CR of retinal and choroidal thickness were 8.37 and 12.20 microns for TMC and 22.24 and 32.40 microns for FCS, and intergrader 95% LoA were 7.37–8.69 and -27.2–27.71 microns for TMC and -34.21–41.93 and -30.46–24.84 for FCS, respectively. Retinal and choroidal thickness showed very good intraobserver reliability for both TMC and FCS (ICC 0.99, LoA 0.98–0.99 in all cases). Intraobserver and interobserver variability for retinal and choroidal thickness was not significantly different for TMC (p = 0.98 and p = 0.90, p = 0.98 and p = 0.91) or FCS (p = 0.97 and p = 0.85, p = 0.78 and p = 0.73), respectively.

**Conclusions:**

Retinal and choroidal thickness in DME eyes can be quantified with good reliability, repeatability and reproducibility using new OCT devices that incorporate swept source technology. The technical advantages of this technology may provide new insights in the understanding of the choroidal changes related with DME.

## Introduction

The management of Diabetic Macular Edema (DME) requires repeated measurements of macular retinal thickness from Optical Coherence Tomography (OCT) devices. Advances in retinal imaging have led to the development of multiple OCT platforms. In the last decade many OCT instruments have become commercially available that incorporate spectral domain (SD-OCT) technology which addresses limitations of time domain (TD-OCT) technology [1]. SD-OCT systems operate using wavelengths of approximately 850nm and scanning speeds between 30,000 and 70,000 A-lines per second [2]. As a consequence, the majority of Eye Clinics have largely transitioned from TD-OCT to SD-OCT instruments, including those participating in clinical trials [1,3,4,5]. Clinical research studies have been directed to rapidly evaluate these new instruments to establish normative databases, assess reproducibility of measurements and develop means of handling data accurately from a variety of instruments, especially in the field of clinical trials. In the context of Diabetic Macular Edema (DME), a specific report from the Diabetic Retinopathy Clinical Research Network (DRCR.net) directed to evaluate the reliability of OCT measurements concluded that a foveal central subfield (FCS) thickness change greater than 10% between visits was accepted as representative of a real change likely beyond measurement error, if measured consistently with the same type of OCT instrument [i.e. Stratus OCT® and Cirrus HD-OCT® (Carl Zeiss Meditec, Dublin, CA), or Spectralis OCT® (Heidelberg Engineering, Heidelberg, Germany)] [1].

More recently, the development of swept-source OCT (SS-OCT) technology, the latest OCT generation, incorporates a tuneable laser source which allows the highest scan speeds on commercially available OCT systems by operating up to 100.000 A-line scans per second. This feature reduces the acquisition time and allows the scanning of wider areas, capturing the optic nerve head and the entire posterior pole up to the vascular arcades in a single 12x9mm image [2]. Moreover, SS-OCT devices use a laser source of a longer wavelength (1050nm) which penetrates deeper in the retinal and choroidal structures than conventional laser sources used in SD-OCT devices. Given these technical advantages, it is sensible to consider that SS-OCT devices will eventually be developed further in the coming years and will replace SD-OCT machines, as it happened before with previous TD-OCT generations.

Nevertheless, advancements in OCT technology require clinical validation. An understanding of the variability of the measurements using SS-OCT devices is mandatory prior to be implemented in a routine clinical setting. In normal eyes, the repeatability and reproducibility of retinal and choroidal thickness measurements using SD-OCT and SS-OCT have been evaluated by few studies [6,7]. Yamashita et al reported good correlation of subfoveal choroidal thickness measurements with three different SD-OCTs, meanwhile Mansouri et al described that the repeatability of automated retinal and choroidal thickness measurements with SS-OCT could be improved after the correction of scan artifacts [8]. Unfortunately however, in the context of diabetic eye disease there is a lack of information in this area. A recent published study has assessed the intrasession repeatability of choroidal thickness measurements obtained using SS-OCT in Type 2 diabetic patients [9]. Hovewer, no study has specifically addressed the reliability of SS-OCT devices in both, retinal and choroidal thickness in DME eyes, where anatomical alterations are common representing a challenge for the segmentation algorithms and therefore the accuracy of the devices. The aim of this study is to evaluate the repeatability and reproducibility of retinal and choroidal thickness measurements using the SS-OCT technology in a cohort of DME eyes.

## Methods

Patients diagnosed with DME were prospectively recruited from Medical Retina clinics at Institut Clinic d´Oftalmologia (ICOF), Hospital Clinic of Barcelona. Inclusion criteria for the diabetic patients included a diagnosis of DME with retinal thickening involving the center of the macula (foveal-involving DME), as per the International Classification [10]. Cases with media opacities that could preclude accurate OCT images adquisition, eyes with refractive error ≥ ±6 diopters, or eyes with structural damage in the macula related to previous treatments (i.e. laser scars) were excluded from the study. All patients were examined by a retina specialist (AS, MF) prior to enrollment in this study. The study protocol was approved by the local Institutional Review Board, namely Ethics Comittee of the Hospital Clinic of Barcelona. Written informed consent was obtained from each patient prior to be included in the study. This research followed the tenets of the Declaration of Helsinki.

### Demographics and clinical data

Clinical data collected included age, gender, diabetes mellitus type, diabetes mellitus treatment type, duration of diabetic disease and comorbilities. Ocular features collected included axial length measured by partial coherence interferometry (IOL Master® 500, Carl Zeiss Meditec, Dublin, CA, USA), refractive status, phakic status, visual acuity and intraocular pressure at the moment of the scan, and previous ocular treatments or intraocular surgery.

### Swept-source Optical Coherence Tomography image acquisition & analysis

SS-OCT images were captured by 2 independent masked experienced image reading center certified examiners (MH, TH) using the Atlantis® DRI-OCT 1 (Topcon Corp., Japan). For the repeatability study, examiner 1 captured 2 consecutive macular cube (12x9mm) image sets with a 10–15 minutes period between each OCT acquisition from each individual eye. For the reproducibility study, examiner 2 captured a second macular cube (12x9mm) image set 20–25 minutes after examiner 1 completed the first examination. Segmentation of retinal and choroidal layers was performed automatically using the device software. In cases of segmentation errors, manual corrections of individual A-scans were performed to fit the boundaries of the compartments of interest (retina: inner limiting membrane, outer layer of RPE; choroid: Bruch´s membrane and outer choroidal border). In each OCT image set, the Early Treatment Diabetic Retinopathy Study (ETDRS) grid was centered in fovea, and measurements of both total macular circle (TMC, ETDRS subfields 1 to 9) and foveal central subfield (FCS, ETDRS subfield 1) retinal and choroidal thicknesses were determined.

### Statistical methods

Clinical demographic and imaging data were analysed with frequency and descriptive statistics. Repeatabilty of measurements or intraobserver variability was addressed with the coefficient of repeatability (CR), which was calculated using the within grader standard deviation (Sw) derived from the intragrader mean square of differences. As defined by Bland and Altman, it is calculated as 1.96 times the SD of the differences between two measurements, as per the formula: CR = 1.96 x √(2Sw^2^) or 2.77 x Sw [11]. To allow comparison with other studies, the CR is also expressed as a percentage of the mean measurement for all retinal and choroidal layers (CR/Mean), with a lower the CR/mean percentage representing greater repeatability within graders. Relative reliability of measurements within graders was evaluated using the intraclass correlation coefficient (ICC).

Reproducibility of measurements or interobserver variability was evaluated using Bland-Altman plots, using the mean thickness determined by each examiner, and the mean difference and the 95% confidence intervals between measurements. Agreement between examiners was evaluated using Bland-Altman analysis, and the 95% limits of agreement (LoA) were calculated, once normality of the measurements differences was confirmed using histograms. All statistical analysis was performed using IBM SPSS Statistics software version 21.0 (IBM Corp., Armonk, NY).

## Results

### Demographics and characteristics of study eyes

A total of 42 eyes from 25 patients with macular edema related to type 2 (n = 24) and type 1 (n = 1) Diabetes Mellitus were included in the study. The baseline characteristics of patients and study eyes are disclosed in Table 1. Mean age of patients was 66.3±12.4 (mean±standard deviation; median 68, interquartile range–IQR- 17) years, with a 52% female preponderance (13/25) and a mean glycosilated hemoglobin (HbA1c) of 7.41±0.5% (median 7.3, IQR 1.3). With regards to ocular conditions, 9.5% of the eyes had no diabetic retinopathy (4/42), 33% had mild non-proliferative diabetic retinopathy (NPDR)(14/42), 35.7% had moderate NPDR (15/42), 9.5% had severe NPDR (4/42) and 11.9% had treated proliferative diabetic retinopathy (PDR)(5/42). Mean VA was 0.45±0.3 logMAR units (median 0.35, IQR 0.45), mean axial length was 23.0±1.3 milimeters (median 23.2, IQR 2.3) and mean refractive error was 0.50±1.4 diopters (median 0.50, IQR 1.93). Thirty-five percent of the study eyes were pseudophakic (35.7%, 15/42) and 64.3% were phakic (27/42). Previous ocular treatments were used in 59.5% of the study eyes (25/42), and included macular laser (14.2%, 6/42), peripheral laser panretinophotocoagulation (9.5%, 4/42), anti-VEGF drugs (33.3%, 14/42), intravitreal triamcinolone (4.7%, 2/42) and intravitreal dexamethasone implant (19.0%, 8/42). At the moment of the scan, 40.4% of the study eyes were treatment-naïve DME eyes (17/42).

**Table 1:** Demographics and baseline characteristics of study eyes.

	Mean, n (%)	SD	Range
**Diabetes Mellitus type**			
- **Type 1 DM**	1 (4)		
- **Type 2 DM**	24 (96)		
**Age**	66.3	12.4	30–83
**Gender (male:female)**	12:13		
**HbA1c (%)**	7.41	0.5	5.7–8.9
**Visual acuity (logMAR)**	0.45	0.3	0–1.3
**Axial length (mm)**	23.0	1.3	21.0–25.6
**Refractive error (diopters)**	0.5	1.4	-4–4
**Phakic status**			
- **Phakic**	27 (64.3)		
- **Pseudophakic**	15 (35.7)		
**Diabetic retinopathy status**			
- **No DR**	4 (9.5)		
- **Mild NPDR**	14 (33.3)		
- **Moderate NPDR**	15 (35.7)		
- **Severe NPDR**	4 (9.5)		
- **Treated PDR**	5 (11.9)		
**Previous ocular treatments**			
- **None**	17 (55.3)		
- **Macular laser**	6 (15.7)		
- **PRP**	4 (10.5)		
- **Anti-VEGF**	12 (31.5)		
- **IVTA**	2 (5.2)		
- **Intravitreal dexamethasone implant**	8 (21.0)		

(DM: Diabetes Mellitus; SD: standard deviation; NPDR: Nonproliferative diabetic retinopathy; PDR: Proliferative diabetic retinopathy).

### Mean retinal and choroidal thickness of study eyes

Mean retinal thickness of the total macular circle (ETDRS subfields 1 to 9) was 339.5±56.3 μm (median 325.0, IQR 87.1) and 339.8±57.1 μm (median 325.7, IQR 89.8) for Observer 1 (first and second measurements)(p = 0.462), and for Observer 2 this was 338.8±55.9 μm (median 328.3, IQR 89.5)(p = 0.306)Table 2. Mean foveal central subfield (ETDRS area 1) thickness was 377.2±101.0 μm (median 342.5, IQR 129) and 377.3±103.1 μm (median 345.5, IQR 126.7)(p = 0.939) for Observer 1 (first an second measurements), and 373.3±99.2 μm (median 343.0, IQR 128)(p = 0.07) for Observer 2.

**Table 2:** Mean retinal and choroidal thickness measurements by observer and measurement.

	Retinal Thickness				Choroidal Thickness			
	Mean	SD	Range	*P value*	Mean	SD	Range	*P value*
**Total Macular Circle (TMC)**								
**Observer 1 –Measurement 1**	339.5	56.3	260.3–480.7	*	213.8	81.8	84.3–455.1	*
**Observer 1 –Measurement 2**	339.8	57.1	262.4–491.2	*0*.*462*	212.8	81.6	88.8–454.6	*0*.*170*
**Observer 2**	338.8	55.9	258.6–477.4	*0*.*306*	213.5	78.9	83.4–433.9	*0*.*868*
**Foveal central subfield (FCS)**								
**Observer 1 –Measurement 1**	377.2	101.0	219.0–680.0	*	226.7	87.0	60.0–475.0	*
**Observer 1 –Measurement 2**	377.3	103.1	213.0–692.0	*0*.*939*	223.8	84.9	69.0–470.0	*0*.*113*
**Observer 2**	373.3	99.2	217.0–700.0	*0*.*076*	229.5	85.0	61.0–470.0	*0*.*204*

(SD: standard deviation; TMC: Total macular circle, ETDRS subfields 1 to 9; FCS: Foveal central subfield, ETDRS central subfield

*: reference)

Mean choroidal thickness of the total macular circle was 213.8±81.8 μm (median 211.5, IQR 105) and 212.8±81.6 μm (median 208.8, IQR 101.8)(p = 0.17) for Observer 1 (first and second measurements), and 213.5±83.4 μm (median 214.5, IQR 107.7)(p = 0.86) for Observer 2. Mean foveal central subfield was 226.7±87.0 μm (median 220, IQR 107) and 223.8±84.9 μm (median 215.5, IQR 95.2)(p = 0.11) for Observer 1 (first and second measurements), and 229.5±85.0 μm (median 233.0, IQR 102.25)(p = 0.20) for Observer 2.

### Repeatability of measurements

**Fig 1:**
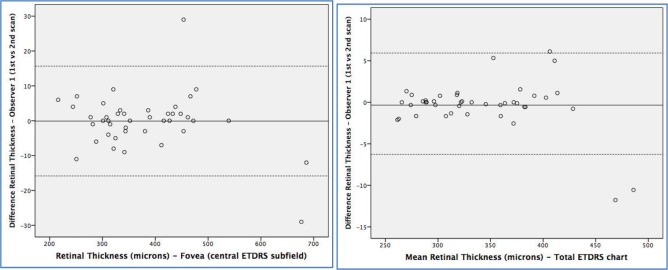
Bland-Altman plots of the different retinal thickness measurements between different scans done by the same observer (repeatability). Foveal central subfield (left) and total macular circle (ETDRS chart, right).

**Fig 2:**
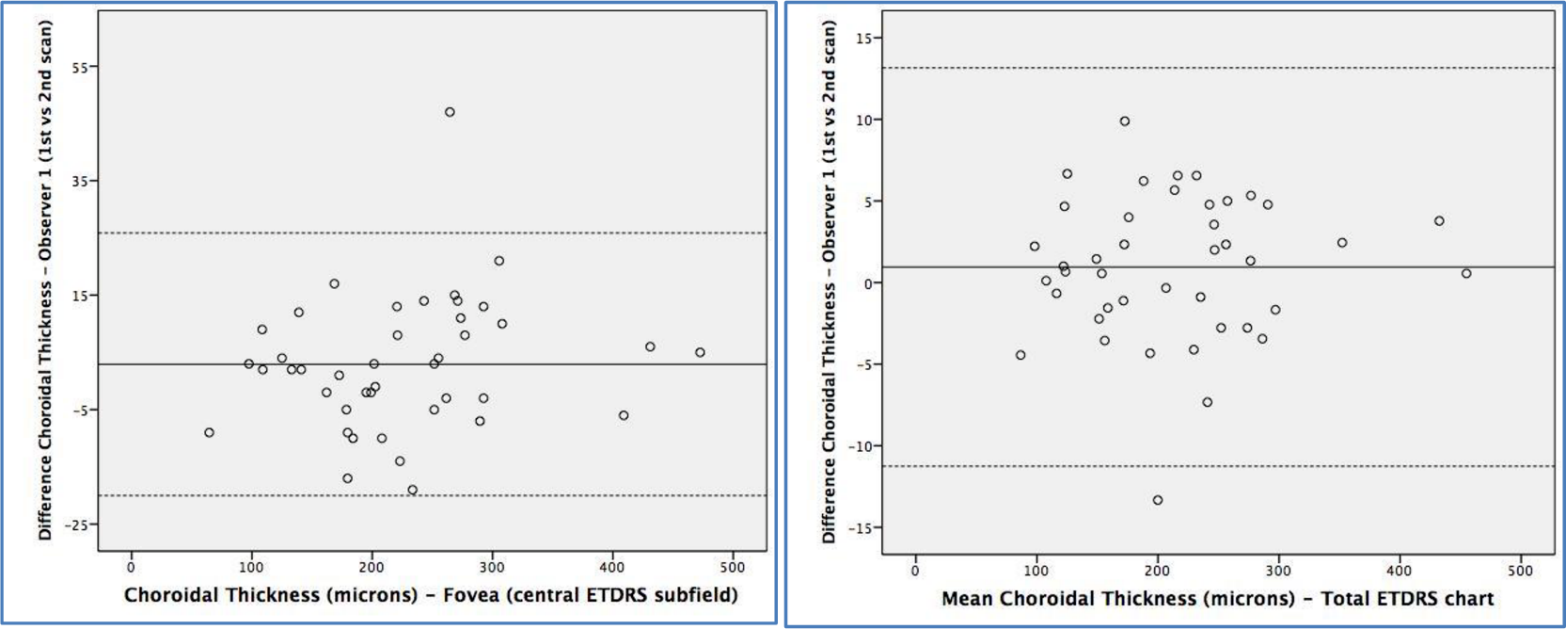
Bland-Altman plots of the different choroidal thickness measurements between different scans done by the same observer (repeatability). Foveal central subfield (left) and total macular circle (ETDRS chart, right).

Thickness measurements in the TMC revealed greater repeatability than FCS, for both retinal and choroidal measurements. The CR for retinal thickness in the TMC was 8.37 μm and in the FCS was 22.24 μm, and for choroidal thickness was 12.20 and 32.40 μm, respectively. The greatest repeatability (lowest CR/mean percentage) was observed in the TMC of the retina (2.46%), followed by the FCS retinal thickness (5.89%) (Table 3). These findings are presented in Bland-Altman plots (Fig 1). Choroidal measurements revealed lower repeatability, with a 12.86% and a 14.38% in the TMC and the FCS, respectively (Fig 2). The reliability of retinal and choroidal thickness measurements was very good (all ICC ≥0.98). The variance ratio (*F* statistic) and the 95% LoA of the intraobserver measurement differences were calculated and were not significantly different for any of the retinal or choroidal thickness parameters (Table 4).

**Table 3:** Repeatability and reliability of retinal and choroidal thickness measurements in patients with Diabetic Macular Edema using swept-source Optical Coherence Tomography.

	CR (microns)	CR/Mean, %	ICC	95% CI
**Retina**				
**TMC**	8.37	2.46	0.99	0.99–0.99
**FCS**	22.24	5.89	0.99	0.99–0.99
**Choroid**				
**TMC**	12.20	12.86	0.99	0.99–0.99
**FCS**	32.40	14.38	0.99	0.98–0.99

(CR: Coefficient of repeatability; ICC: Intraclass correlation coefficient; CI: Confidence interval; TMC: Total macular circle, ETDRS subfields 1 to 9; FCS: Foveal central subfield, ETDRS central subfield).

**Table 4:** The variance ratio (F statistic) of intragrader and intergrader differences in retinal and choroidal measurements.

	Intragrader		Intergrader	
	Thickness measurements		Thickness measurements	
	*F* Test	P Value	*F* Test	P Value
**Retina**				
**TMC**	0.97	0.987	1.01	0.901
**FCS**	0.96	0.978	1.03	0.856
**Choroid**				
**TMC**	1.00	0.983	1.07	0.918
**FCS**	1.04	0.783	1.04	0.738

(TMC: Total macular circle, ETDRS subfields 1 to 9; FCS: Foveal central subfield, ETDRS central subfield).

### Reproducibility of measurements

**Fig 3:**
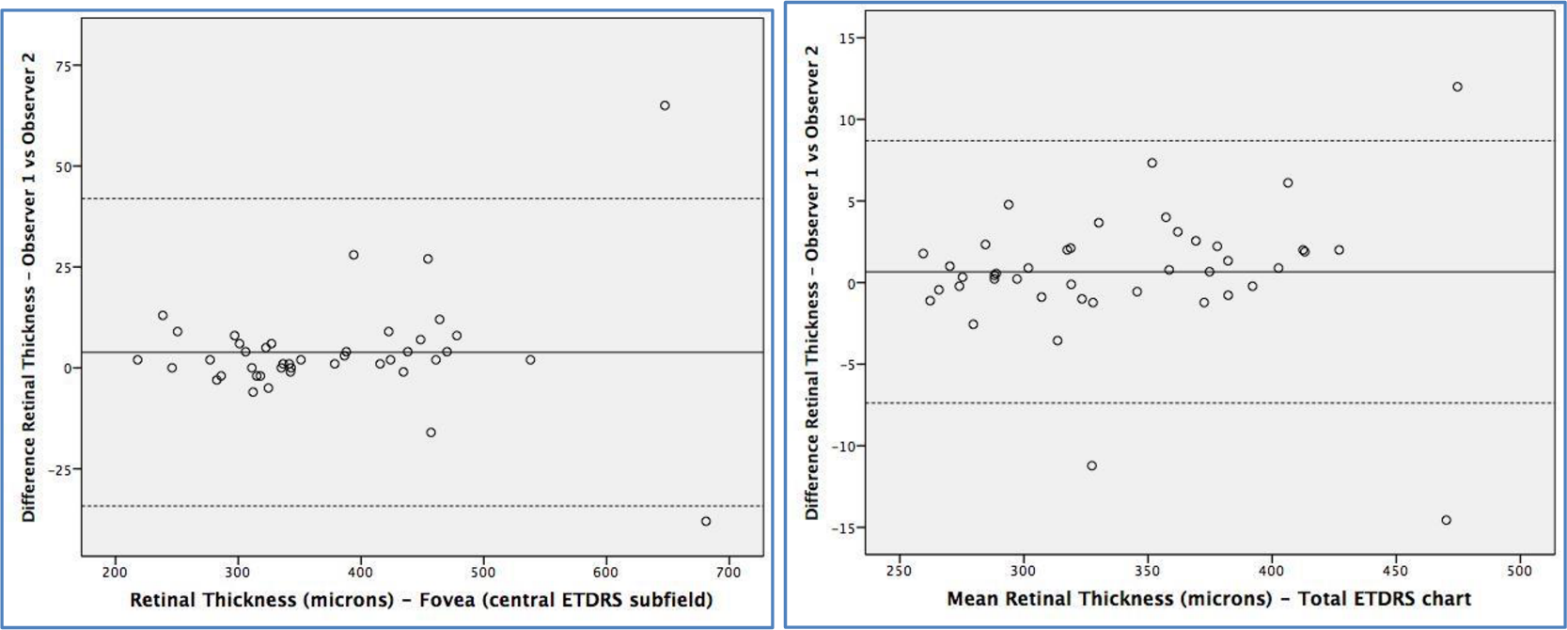
Bland-Altman plots of the different retinal thickness measurements between different observers (reproducibility). Foveal central subfield (left) and total macular circle (ETDRS chart, right).

**Fig 4:**
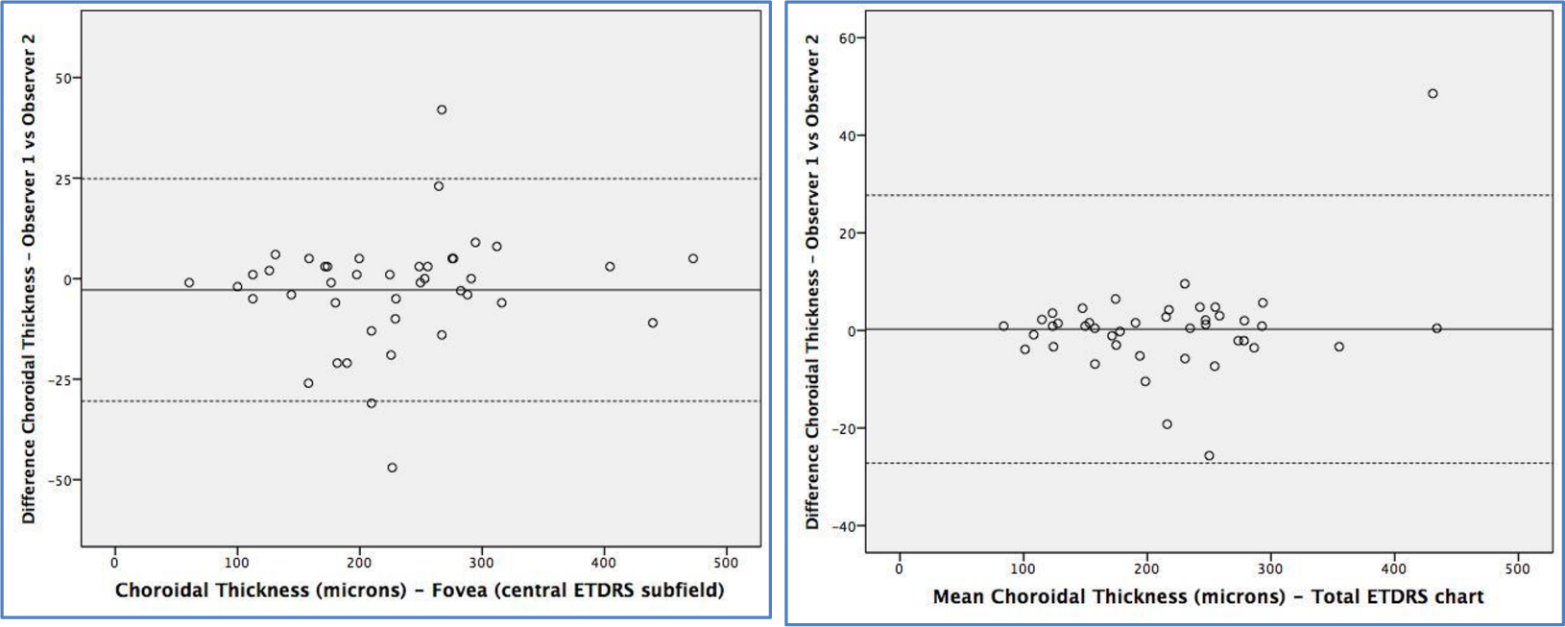
Bland-Altman plots of the different choroidal thickness measurements between different observers (reproducibility). Foveal central subfield (left) and total macular circle (ETDRS chart, right).

The interobserver mean difference in retinal and choroidal thickness together with the 95% confidence intervals is presented in Table 5 and represented graphically in Bland-Altman plots (Figs 3 and 4). Mean difference in retinal thickness between examiners 1 and 2 was 0.65 μm in the TMC and 3.85 μm in the FCS (95% CI -0.62–1.93, and -0.42–8.14, respectively). Differences in choroidal thickness were 0.25 μm in the TMC and -2.80 in the FCS (95% CI -2.83–3.34, and -7.20–1.58 respectively). Limits of agreement at 95% for retinal thickness were 16.06 and 76.14 μm in the TMC and FCS respectively, and for choroidal thickness were 54.91 and 55.3 μm in the same areas. The variance ratio (*F* statistic) and the 95% LoA of the interobserver measurement differences were calculated and were not significantly different for any of the retinal or choroidal thickness parameters (Table 4).

**Table 5:** Reproducibility of retinal and choroidal thickness measurements in patients with Diabetic Macular Edema using swept-source Optical Coherence Tomography.

	Interobserver mean difference (microns)	95% CI (microns)	95% Limits of agreement (microns)
**Retina**			
**TMC**	0.65	-0.62–1.93	16.06
**FCS**	3.85	-0.42–8.14	76.14
**Choroid**			
**TMC**	0.25	-2.83–3.34	54.91
**FCS**	-2.80	-7.20–1.58	55.3

(CI: Confidence interval; TMC: Total macular circle, ETDRS subfields 1 to 9; FCS: Foveal central subfield, ETDRS central subfield).

## Discussion

Our results show that retinal and choroidal thickness in DME eyes can be quantified with good reliability, repeatability and reproducibility using SS-OCT. This data offers preliminary evidence to support the use of SS-OCT in the management of DME, and evaluates its ability to objectively detect and quantify the severity of macular edema. These findings appear relevant as accurate detection of quantitative OCT changes in DME eyes is capital to guide retreatment in both routine clinical care and clinical trials.

The two main technical advantages of SS-OCT are the faster scanning speed, which allows the capture of a single image covering a larger area than SD-OCT devices, and the deeper penetration of the laser beam, which allows a better delineation of the posterior edge of the choroid [2]. Both features appear relevant in the management of DME. First, it is a relatively common scenario to see DME cases in which the edematous area extends beyond the standard 6x6 mm cube, making the management of these cases difficult as response to treatment can only be evaluated in the scanned area. Larger macular cubes covering wider areas (12x9mm) may help the clinician in the decision making process in such cases, as scans could be more sensitive to detect improvements after treatment or early changes during follow up. Second, there is an increasing interest in the study of the choroidal changes seen in diabetic eye disease, so-called diabetic choroidopathy, where several authors have recently identified some OCT signs with deep penetration techniques [12–14]. In the near future, the advantage of SS-OCT to obtain more detailed images of the posterior choroid could allow a better understanding of its role in the pathophysiology of diabetic eye disease.

We observed a very good intragrader repeatability of retinal thickness measurements with the SS-OCT device, especially in the total macular circle (TMC 8.37 μm, FCS 22.24 μm). There is few data in the literature about such parameter in diabetic eyes using SS-OCT, but there are some reports evaluating the repeatability of retinal thickness measurements using SD-OCT devices, presented in Table 6. In 2008, Forooghian et al. [15] reported a very good intrasession repeatability for FCS with Stratus and Cirrus (17.9 μm and 19.0 μm), showing an even better coefficient of repeatability than our study using SS-OCT (22.2 μm). However, posterior studies have not achieved such outcomes. Bressler et al [1]. reported a lower repeatability for FCS measured with Stratus and the RTVue device (Optovue, Fremont, California, USA) (33.0 μm and 40.0 μm, respectively), and similarly Sim et al [16]. published a higher CR using Spectralis (49.0 μm). As we observed in our series, in the latter they found that measurements from the TMC showed better repeatability and agreement compared with the FCS alone [16]. Whereas direct comparisons between studies cannot be made due to differences in methodology and study cohorts, the outcomes reported in this series suggest that repeatability of SS-OCT measurements seems to be slightly better than previous published data for SD-OCT (with the exception of the study by Forooghian et al) [15].

**Table 6:** Coefficients of reproducibility of previous studies in Diabetic Macular Edema using time domain, spectral domain and current study with Swept source Optical Coherence Tomography (OCT) in the ETDRS central subfield.

Author, Year		Device	N	CR (microns)		CR/Mean, %	
				Retinal Thickness	Choroidal Thickness	Retinal Thickness	Choroidal Thickness
Forooghian, 2008	DME	Stratus ®, Zeiss	33	17.9	-	2.63	-
	DME	Cirrus ®, Zeiss		19.0	-	2.42	-
Wolf-Schnurrbusch, 2009	Controls	Stratus ®, Zeiss	20			3.33	
		Cirrus ®, Zeiss				3.09	
		Spectralis ®, Heidelberg				0.46	
		OCT/SLO ®, Heidelberg				2.23	
		RTVue 100 ®, Optovue				2.77	
		SOCTCopernicus®, Reichert				3.5	
Vujosevic, 2012	DR	RS-3000 ® Nidek	102*	-	28.8	-	-
Sim, 2013	DME	Spectralis ®, Heidelberg	51	49.0	48.3	18.1	19.6
Fiore, 2013	Controls	Spectralis ®, Heidelberg	12			2.2	
	DME	Spectralis ®, Heidelberg	21			2.4	
Bressler, 2015	DME	Stratus ®, Zeiss	309	33.0	-	10	-
	DME	RTVue 100 ®, Optovue		40.0	-	16	-
***This study***	***DME***	***Atlantis ®*, *Topcon***	***42***	***22*.*24***	***32*.*4***	***5*.*89***	***14*.*38***

(CR: Coefficient of repeatability

*: Diabetic eyes).

Our study also examined the intergrader variability of retinal thickness measurements. The results have revealed good reproducibility of measurements with the SS-OCT device, with a mean intergrader difference in retinal thickness of 0.65 μm in the TMC and 3.85 **μ**m in the FCS. There is limited data in the literature about reproducibility of SD-OCT devices in both controls and DME eyes. In normal eyes, Wolf-Schnurrbusch U et al [7]. compared six different OCT devices and reported good reproducibility values for all of them (ranging from 0.46% to 3.5%, Table 6), suggesting that the small discrepancies were due to retinal segmentation algorithms between the machines. In this study, Spectralis HRA-OCT showed the best results, with a significantly lower variation between measurements compared to the Stratus OCT (which was used as reference) [7]. In contrast to this data in normal eyes, we have observed a lower reproducibility in DME eyes with SS-OCT (5.89%). However, this figure is better than the data reported by two other recent studies in DME eyes using Spectralis OCT, one by Sim et al. which reported a CR of 18.1% and another one by Fiore et al. which showed a CR of 3.02% in healthy eyes and 8.18% in DME [16,17]. If confirmed in future series, these results suggest that the reproducibility of retinal thickness measurements using SS-OCT may be higher than SD-OCT devices in DME eyes.

Few studies have examined the reproducibility of choroidal thickness measurements assessed by OCT. A recent study in a Japanese cohort of normal eyes reported good reproducibility of foveal choroidal thickness measurements obtained with three different SD-OCT devices [6]. In DME eyes, to date two previous SD-OCT studies and one recent SS-OCT study have been reported [9,16,18]. Using SD-OCT, Vujosevic et al [16]. showed good reproducibility in subfoveal choroidal thickness measurements (28.8 μm), whereas Sim et al [18]. showed moderate intragrader repeatability in both the macula and the fovea (TMC 26.9 μm, FCS 48.3 μm, Table 6). Using SS-OCT, Abadia et al [9]. obtained intraclass correlation coefficient (ICCs) values close to one in all choroidal locations in the whole sample and in both healthy and diabetic groups, and no significant differences (p>0.05) were found in the intratest repeatability of any choroidal measurement between healthy controls and Type 2 diabetic (T2D) patients. According to that study authors, these results confirmed the low variability of choroidal thickness measurements acquired with SD-OCT and SS-OCT [9].

Interestingly, we observed a similar CR than the Vujosevic study but greater repeatability (TMC 12.20 μm, FCS 32.40 μm) than the Sim study, consistently with the data reported above for retinal measurements. These findings may suggest that the reproducibility of choroidal measurements appears to be device-specific, and not only related to the type of OCT (SD-OCT and SS-OCT).

A recent published study from Abadia et al [19]. found significant differences in mean subfoveal choroidal thickness measurements between healthy eyes (228.1±78.8 μm, n = 71) and DME eyes (183.5±72.9 μm, n = 48) (p = 0.002), and they concluded that choroidal thickness was significantly reduced in T2D patients compared to healthy controls [19]. Interestingly, no differences were found between DME and no DME patients within the T2D patients. In our study, the mean choroidal thickness of the foveal central subfield was 226.7±87.0 μm (median 220, IQR 107) and 223.8±84.9 μm (median 215.5, IQR 95.2) (p = 0.11) for Observer 1 (first and second measurements), and 229.5±85.0 μm (median 233.0, IQR 102.25) (p = 0.20) for Observer 2. However, we did not compare this findings with a healthy control group.

This study has a number of limitations. First, the small sample size may have contributed to overestimate the reliability of the measurements, which may have been challenged in a larger series of cases with arguably wider variations in the measurements obtained. Second, as previously mentioned, no control group has been included to evaluate the measurements obtained in the present study with those obtained in healthy eyes. Another limitation is that, segmentation of the scans was automatically performed by the device software but segmentation errors were corrected manually to fit the boundaries of the regions of interest when required, which only reflect ideal conditions in the research setting. This implies that the outcomes reported in this study cannot be directly translated to the commercial versions of the device routinely used in clinical care. This consideration is especially important in the context of DME, where segmentation errors are frequent due to hard exudates, epiretinal membranes, optical opacities or serous detachments [20]. And finally, the eyes included in the study were selected according to some specific inclusion and exclusion criteria, and the observed outcomes may not be applicable to all DME eyes (i.e. DME eyes with macular scars from previous laser treatments, frequently seen in the clinical setting).

In conclusion, this study reports that the retinal and choroidal thickness in DME eyes can be quantified with good reliability, repeatability and reproducibility using a SS-OCT device. The technical advantages of SS-OCT technology may provide additional benefits in the evaluation of macular diseases compared to SD-OCT machines, mainly related to a higher scanning speed which allows the image capture of larger areas and a greater penetration in the deeper retinal and choroidal layers. We believe that in the near future this technology may become the gold standard technique in the evaluation of DME.

## Supporting information

S1 File Clinical data SS-OCT in DME patients. (XLSX)
